# Bayesian networks for predicting clinical outcomes in COVID-19 patients: A retrospective study in a resource-limited setting

**DOI:** 10.1371/journal.pone.0343096

**Published:** 2026-03-13

**Authors:** Tombolaza Canut Filamant, Angelo Fulgence Raherinirina, André Totohasina

**Affiliations:** 1 Thematic Doctoral School Science, Culture, Society and Development, University of Toamasina, Toamasina, Madagascar; 2 Research Center for Mathematics Education, University of Fianarantsoa, Fianarantsoa, Madagascar; 3 Department of Mathematics and Informatics, University of Antsiranana, Antsiranana, Madagascar; Christian Medical College Vellore, INDIA

## Abstract

**Background:**

The COVID-19 pandemic has highlighted the critical need for robust, interpretable predictive models to guide clinical decision-making for hospitalized patients, particularly in resource-limited settings. While machine learning approaches have achieved high predictive performance, many lack the transparency required for clinical adoption. Bayesian networks provide a rigorous mathematical framework for modeling medical uncertainty and complex causal relationships while maintaining clinical interpretability.

**Objective:**

To develop and validate an interpretable Bayesian network model for predicting clinical outcomes in hospitalized COVID-19 patients, including severity, complications, and mortality, and to compare its performance with existing predictive approaches. The model specifically addresses the needs of resource-limited settings where both interpretability and performance are critical.

**Methods:**

This retrospective cohort study analyzed 124 hospitalized COVID-19 patients at Tanambao I University Hospital, Madagascar (March 2020–March 2022). Given limited genomic sequencing capacity during the study period, specific SARS-CoV-2 variants infecting individual patients could not be confirmed. After descriptive and statistical implicative analysis, we constructed a Bayesian network integrating predictive variables organized in three levels: input variables (demographic, vital signs, biological, radiological), intermediate variables (clinical scores), and target variables (severity, complications, evolution, death). Parameter learning used maximum likelihood estimation with Laplace smoothing. Model performance was evaluated using stratified 10-fold cross-validation and compared against logistic regression, random forest, and support vector machine approaches.

**Results:**

The Bayesian network model exhibited strong diagnostic performance, with an AUC of 0.95 (95% CI: 0.91–0.99) for death prediction, 0.94 (95% CI: 0.90–0.98) for severe outcomes, and 0.93 (95% CI: 0.89–0.97) for unfavorable progression. Stratified 10-fold cross-validation yielded a mean accuracy of 0.85±0.03. The model outperformed logistic regression (AUC 0.89), random forest (AUC 0.91), and SVM (AUC 0.87) while maintaining superior interpretability. Sensitivity analysis identified qSOFA score (mutual information: 0.342), SpO_2_ levels (0.298), and respiratory distress (0.276) as the most influential variables. Robustness testing showed prediction stability under parameter perturbations of ±15%, with variation remaining below 8%.

**Conclusions:**

Bayesian networks constitute a promising tool for COVID-19 outcome prediction in resource-limited settings, combining competitive predictive performance with essential clinical interpretability. The probabilistic approach enables rigorous uncertainty quantification critical for medical decision-making. While the model achieved excellent performance on our cohort, external validation across different populations and SARS-CoV-2 variants is needed to establish broader generalizability.

## Introduction

The COVID-19 pandemic has posed unprecedented challenges to healthcare systems worldwide, necessitating robust predictive tools to optimize the management of hospitalized patients [1]. The significant variability in clinical evolution, ranging from asymptomatic forms to fatal multi-organ failure, underscores the complexity of the underlying pathophysiological mechanisms [2]. Early and accurate prediction of adverse outcomes is critical for resource allocation, particularly in settings with limited intensive care capacity.

### The challenge of COVID-19 outcome prediction

Numerous predictive models have been developed for COVID-19 prognosis. Wynants et al. [3] systematically reviewed 232 prediction models for COVID-19 diagnosis and prognosis, finding that while many reported high discrimination (C-index ranging from 0.65 to >0.99), most suffered from high risk of bias, lack of external validation, and poor reporting standards. This highlighted the need for methodologically rigorous approaches that balance predictive performance with clinical interpretability and generalizability. Machine learning approaches have shown promise in COVID-19 prediction. Yan et al. [4] developed an interpretable decision tree model using only three blood biomarkers in a Wuhan cohort (N = 485), achieving AUC > 0.90 in both development and validation cohorts. Their emphasis on minimal feature use and interpretability demonstrated that simpler models could achieve competitive performance while remaining clinically actionable. Banoei et al. [5] developed a machine learning-based mortality model validated across multiple centers, achieving training AUC ≈ 0.94 and validation AUC ≈ 0.90, significantly outperforming traditional clinical scores like APACHE II and SOFA in the same dataset. Traditional predictive models in medicine, often based on logistic regression approaches, present important limitations in the COVID-19 context: difficulty in modeling complex variable interactions, absence of uncertainty quantification, and lack of decision interpretability [3]. While more sophisticated machine learning methods can capture complex patterns, many operate as “black boxes”, limiting their clinical acceptance and potentially obscuring important causal relationships [6].

### Bayesian networks as an interpretable alternative

Bayesian networks, introduced by Pearl [7], offer a rigorous mathematical framework for modeling complex causal relationships and quantifying uncertainty associated with predictions. Unlike “black box” models, they allow transparent interpretation of decision mechanisms through explicit representation of conditional dependencies between variables—a crucial aspect in clinical medicine [8,9]. The graphical structure of Bayesian networks makes the reasoning process visible to clinicians, facilitating trust and adoption. Several studies have successfully applied Bayesian networks to medical prediction tasks. Kahn et al. [10] demonstrated their effectiveness for ICU outcome prediction, achieving competitive performance while providing interpretable probabilistic reasoning. Orphanou et al. [11] used dynamic Bayesian networks for cardiovascular risk prediction, highlighting their ability to model temporal evolution—a feature potentially valuable for COVID-19 progression modeling. Statistical implicative analysis, developed by Gras [12], constitutes a data analysis method particularly adapted to identifying quasi-exact association rules in contexts where deterministic relationships are rare. This approach is particularly relevant in medicine, where relationships between variables are often probabilistic rather than deterministic. By identifying robust implicative relationships, this method can guide the structure learning phase of Bayesian network construction, potentially improving model quality and interpretability.

### The context of low-and middle-income countries

In low- and middle-income countries (LMICs), where healthcare resources are often limited, the need for accurate, interpretable, and computationally efficient predictive models is even more critical [13]. In such settings, models must not only predict accurately but also provide transparent reasoning that can guide clinical decisions by non-specialist healthcare workers.

The interpretability-performance trade-off is particularly important in resource-limited settings. While complex machine learning models may achieve marginally better performance, their lack of interpretability can limit adoption and trust among clinicians who must make high-stakes decisions with limited resources. Models that explicitly represent causal relationships and quantify uncertainty may be more valuable in such contexts, even if their performance is comparable rather than superior to black-box alternatives.

### Study objectives and contributions

The objective of this study is to develop and validate a Bayesian network model for predicting clinical outcomes in hospitalized COVID-19 patients, using a rigorous methodological approach combining descriptive analysis, statistical implicative analysis, and probabilistic modeling. Our specific contributions include:

1. Development of an interpretable Bayesian network model tailored for resource-limited settings, using commonly available clinical variables
2. Systematic comparison with existing machine learning approaches (logistic regression, random forest, SVM) on the same dataset
3. Integration of statistical implicative analysis to guide structure learning and identify robust causal relationships
4. Comprehensive evaluation including cross-validation, sensitivity analysis, and robustness testing
5. Identification of critical predictive pathways that align with clinical knowledge and can guide intervention strategies

While our model was developed on a single-center cohort during a specific period (March 2020–March 2022), we provide detailed methodological documentation to facilitate external validation and adaptation to other settings. We explicitly discuss limitations related to data quality and generalizability, and propose directions for future work.

## Materials and methods

### Study setting and population

This retrospective study was conducted at Tanambao I University Hospital in Antsiranana, Madagascar, over a 24-month period (March 2020 to March 2022). The hospital serves as a referral center for northern Madagascar, with a 40-bed COVID-19 unit including 8 intensive care beds.

#### Temporal context and variant considerations.

The study period (March 2020–March 2022) spanned multiple SARS-CoV-2 epidemic waves in Madagascar. However, systematic variant sequencing was not routinely performed at our institution or in Madagascar during this period. Like most African countries, Madagascar had severely limited genomic sequencing capacity during 2020–2022, with only sporadic sequencing capability and minimal contribution to global genomic databases [14,15]. Consequently, we cannot definitively confirm which specific SARS-CoV-2 variants were present in our cohort. This represents a significant limitation for model interpretation. Different SARS-CoV-2 variants exhibit substantially different clinical characteristics [16,17], which could materially impact the relationships between predictors and outcomes.

#### Inclusion and exclusion criteria.

Patients were included if they were: (1) hospitalized with RT-PCR or antigen-confirmed COVID-19 infection, or clinically compatible cases with typical chest imaging findings; (2) aged > 18 years; (3) had complete medical records allowing follow-up until discharge or death. Exclusion criteria comprised: (1) asymptomatic cases; (2) hospitalization solely for isolation purposes; (3) discharge against medical advice; (4) transfer to another hospital; and (5) incomplete records preventing complete variable assessment.

After applying these criteria, 124 patients were retained from 205 initially assessed, yielding an inclusion rate of 60.5%. The study was approved by the institutional ethics committee (reference: IEC-TANU-2022-045) and conducted according to the Declaration of Helsinki principles. Given the retrospective nature of the study using anonymized medical records, the requirement for informed consent was waived by the ethics committee in accordance with Malagasy national research regulations.

### Data collection and quality assessment

Data were collected from clinical records and entered into Epi Info 7.2.2 by trained research assistants. The anonymized medical data were accessed for research purposes on 26/03/2022. Variables analyzed included demographic characteristics, clinical presentations, comorbidities, vital signs, laboratory findings, radiological assessments, and clinical outcomes.

### Outcome definitions

Clinical severity was assessed using the quick Sequential Organ Failure Assessment (qSOFA) score [18]. We defined the following outcomes:

- **Severity:** Classified as severe if qSOFA ≥ 2 or presence of major complications (shock, respiratory failure requiring mechanical ventilation, or multi-organ failure)
- **Complications:** Any of the following during hospitalization: respiratory deterioration, diabetic decompensation, cardiac decompensation, shock, altered consciousness, acidocetosis, renal failure, secondary infection, or cardiac arrhythmia
- **Unfavorable Progression:** Defined as worsening clinical status requiring escalation of care or development of new major complications
- **Death:** In-hospital mortality from any cause

All outcome assessments were based on objective clinical criteria documented in medical records, minimizing subjective bias.

### Statistical Implicative Analysis (SIA)

Statistical implicative analysis was employed to identify significant directional implication rules between observed variables and clinical outcomes. This method quantifies the strength and statistical significance of quasi-deterministic relationships, making it particularly suitable for identifying robust predictors in medical data where perfect determinism is rare.

#### Data coding.

For the application of implicative statistics, we performed binary coding of continuous variables according to clinically relevant thresholds established by international guidelines and expert consensus (Table 1). These thresholds represent clinically meaningful cutoffs used in routine practice.

**Table 1:** Binary coding of continuous variables for implicative analysis.

Variable	Threshold	Clinical Rationale
Age	> 65 years	Established risk factor for severe COVID-19
SpO_2_	< 90%	Hypoxemia threshold for intervention
Respiratory rate	> 24 bpm	Tachypnea indicating respiratory distress
Temperature	> 38.5°C	High fever threshold
CRP	> 50 mg/L	Moderate-to-severe inflammation
Creatinine	> 110 μmol/L	Acute kidney injury threshold
Glucose	> 7.0 mmol/L	Hyperglycemia
White blood cells	> 10 G/L	Leukocytosis
Neutrophils	> 7 G/L	Neutrophilia
Lymphocytes	< 1 G/L	Lymphopenia
D-dimers	> 500 ng/mL	Thrombotic risk threshold

#### Target variables.

Primary target variables for implicative analysis:

- DEATH: 1 if death, 0 if survival
- SEVERITY: 1 if qSOFA ≥ 2 or major complications, 0 otherwise

#### Implication intensity and statistical framework.

The implication intensity ϕ(a,b) between two binary variables a and b measures the degree to which a implies b beyond what would be expected by chance. It is defined by:


$$\phi (a,b)=\frac{1}{\sqrt{2\pi }}\int_{t(a,b)}^{+\infty }e^{-\frac{u^{2}}{2}}du$$ (1)

where the critical threshold t(a,b) is calculated as:


$$t(a,b)=\frac{n_{ab}-\frac{n_{a}\cdot n_{b}}{n}}{\sqrt{\frac{n_{a}\cdot n_{b}\cdot (n-n_{a})\cdot (n-n_{b})}{n^{3}}}}$$ (2)

Here, n is the total sample size, $n_{a}$ is the number of individuals satisfying a, $n_{b}$ is the number satisfying b, and $n_{ab}$ is the number satisfying both a and b. The threshold t(a,b) quantifies how much the observed co-occurrence $n_{ab}$ deviates from the expected value under independence, normalized by the standard deviation.

#### Implication index.

The implication index q(a,b) represents the confidence level of the implication:


q(a,b)=1−ϕ(a,b) (3)

This index varies between 0 and 1, with values close to 1 indicating strong implication. We set a significance threshold at q(a,b)≥0.95, corresponding to α=0.05.

#### Contribution index.

To measure the practical importance of an implication, we use the contribution index c(a,b):


$$c(a,b)=\frac{n_{ab}-\frac{n_{a}\cdot n_{b}}{n}}{\sqrt{\frac{n\cdot n_{a}\cdot n_{b}\cdot (n-n_{a})\cdot (n-n_{b})}{n^{3}}}}$$ (4)

This index quantifies the strength of association while adjusting for marginal frequencies, providing a measure of effect size complementary to the statistical significance captured by q(a,b).

#### Implicative graph construction.

**Fig 1:**
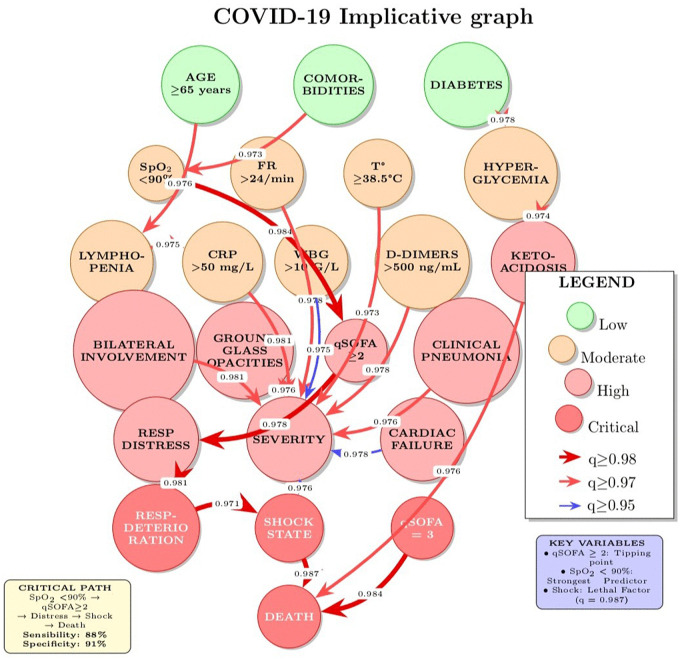
Implication graph of critical paths leading to severity and death.

The implicative graph (Fig 1) represents significant causal relationships as a directed network where:

- Nodes represent binary variables
- Directed edges represent statistically significant implication rules (q≥0.95)
- Edge weights correspond to the implication strength (q index)
- Edge thickness is proportional to implication strength for visual interpretation

The graph construction algorithm proceeds as follows:

1. Compute implication indices q(a,b) for all pairs of binary variables
2. Filter implications based on the significance threshold (q≥0.95)
3. Remove redundant edges using transitive reduction to identify direct relationships
4. Identify critical paths from risk factors to target outcomes

### Bayesian network construction

#### Structure learning.

The Bayesian network structure was defined through a hybrid approach combining:

1. **Statistical implicative analysis:** Robust implicative relationships (q≥0.95) identified in the previous step were used to guide edge inclusion
2. **Constraint-based learning:** We applied the PC algorithm [19] to identify additional conditional independencies in the data
3. **Clinical expert knowledge:** Domain experts reviewed the proposed structure to ensure biological plausibility and remove spurious relationships
4. **Structure validation:** The final structure was validated using Bayesian Information Criterion (BIC) and through cross-validation of predictive performance

**Fig 2:**
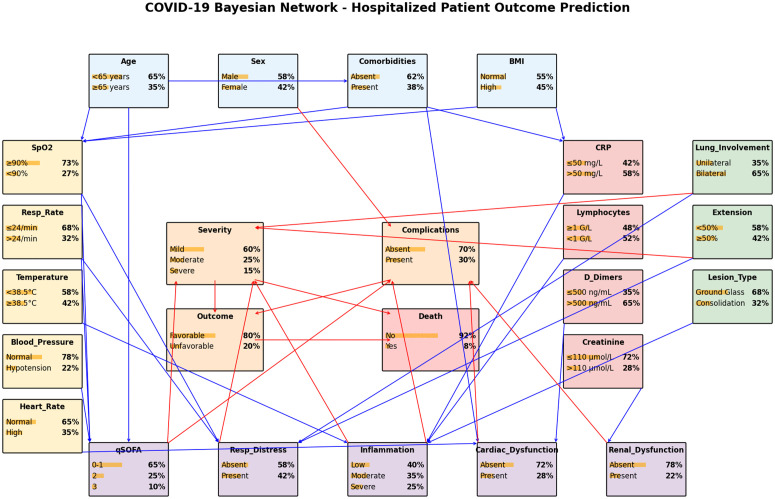
Causal structure of the COVID-19 Bayesian network model.

The resulting network consists of three hierarchical levels (Fig 2)

- **Input variables (Level 1):** Demographic characteristics, vital signs, laboratory values, and radiological findings
- **Intermediate variables (Level 2):** Clinical scores (qSOFA) and pathophysiological states (respiratory distress, systemic inflammation)
- **Target variables (Level 3):** Clinical outcomes (severity, complications, evolution, death)

#### Parameter learning.

Conditional probability tables (CPTs) were estimated from observed data using maximum likelihood estimation with Laplace smoothing:


$$P(X_{i}=x_{i}|Parents(X_{i})=\pi_{i})=\frac{N(x_{i},\pi_{i})+\alpha }{N(\pi_{i})+\alpha \cdot |Val(X_{i})|}$$ (5)

where $N(x_{i},\pi_{i})$ is the number of occurrences of $X_{i}=x_{i}$ with $Parents(X_{i})=\pi_{i}$, $N(\pi_{i})$ is the total number of occurrences of $Parents(X_{i})=\pi_{i}$, α is the smoothing parameter (set to 1), and $\left|Val(X_{i})\right|$ is the number of possible states of $X_{i}$. Laplace smoothing prevents zero probabilities for unobserved configurations, improving model robustness.

#### Inference algorithms.

For probability calculations, we used variable elimination for exact inference when computationally feasible, and Gibbs sampling for approximate inference in cases of high computational complexity. All algorithms were implemented using the bnlearn package [20] in R version 4.2.0.

##### Exact inference.

Variable elimination computes marginal probabilities through systematic elimination of variables, with computational complexity dependent on the network’s treewidth:


$$Complexity=O(n\cdot d^{w+1})$$ (6)

where n is the number of variables, d is the maximum domain size, and w is the treewidth of the graph. Our network’s modest treewidth (maximum 4) allowed efficient exact inference for most queries.

##### Approximate inference.

For scenarios requiring repeated inference under uncertainty (e.g., robustness testing), we employed Gibbs sampling with 10,000 samples and a burn-in period of 1,000 iterations. Convergence was assessed using the Gelman-Rubin diagnostic [21].

### Model evaluation

#### Cross-validation strategy.

Model performance was evaluated using stratified 10-fold cross-validation to ensure balanced representation of outcomes across folds. Stratification was performed on the primary outcome (death), ensuring approximately equal proportions of deceased and surviving patients in each fold. This approach provides unbiased estimates of generalization performance while maximizing use of limited data.

For each fold:

1. 90% of data were used for training (structure learning and parameter estimation)
2. 10% were held out for testing
3. The process was repeated 10 times with different train-test splits
4. Performance metrics were averaged across folds

#### Performance metrics.

We calculated the following metrics for each outcome:

- **Area under the ROC curve (AUC):** Primary metric for discrimination ability
- **Precision:** Positive predictive value
- **Recall (Sensitivity):** True positive rate
- **Specificity:** True negative rate
- **F1-score:** Harmonic mean of precision and recall
- **Positive predictive value (PPV):** Proportion of positive predictions that were correct
- **Negative predictive value (NPV):** Proportion of negative predictions that were correct

Confidence intervals (95%) for AUC were computed using DeLong’s method [22].

#### Variable importance.

Variable importance was quantified using mutual information I(X;Y) between each predictor X and the target outcome *Y*:


$$I(X;Y)=\sum_{x\in X}\sum_{y\in Y}P(x,y)\log\frac{P(x,y)}{P(x)P(y)}$$ (7)

Higher mutual information indicates stronger predictive value. This metric is particularly appropriate for Bayesian networks as it naturally captures both linear and non-linear dependencies.

#### Robustness testing.

We assessed model robustness through multiple complementary analyses:

1. **Parameter perturbation:** CPT parameters were perturbed by 5%, 10%, and 15% using Gaussian noise, and prediction variability was quantified
2. **Input noise injection:** Input variables were corrupted with various noise levels (5–15%) to simulate measurement error common in LMIC settings
3. **Missing data sensitivity:** We randomly removed 10%, 20%, and 30% of input variables to assess degradation of performance
4. **Bootstrap validation:** 1,000 bootstrap samples were generated to assess stability of parameter estimates and performance metrics

For each robustness test, we computed a robustness score:


$$R=1-\frac{\sigma_{pred}}{\mu_{pred}}$$ (8)

where $\sigma_{pred}$ is the standard deviation of predictions under perturbation and $\mu_{pred}$ is the mean prediction. Higher scores indicate greater robustness.

### Comparison with other approaches

To contextualize our model’s performance, we trained and evaluated three commonly used machine learning models on the same dataset:

- **Logistic Regression:** L2-regularized with hyperparameters tuned via grid search
- **Random Forest:** 500 trees with maximum depth optimized via cross-validation
- **Support Vector Machine:** RBF kernel with cost and gamma parameters tuned via grid search

All models used identical train-test splits and evaluation metrics, ensuring fair comparison. Feature selection was performed separately for each model using recursive feature elimination. Models were implemented using scikit-learn [23] version 1.0.2.

### Statistical analysis

Descriptive statistics were calculated for all variables. Continuous variables were expressed as median [interquartile range] due to non-normal distributions confirmed by Shapiro-Wilk tests. Categorical variables were expressed as frequencies and percentages. Comparisons between survivors and non-survivors were performed using Mann-Whitney U test for continuous variables and Fisher’s exact test for categorical variables, with Bonferroni correction for multiple comparisons. Statistical significance was set at p<0.05 after correction. All analyses were performed using R version 4.2.0 with the following packages: bnlearn [20], pROC [24], caret [25], and ggplot2 [26].

## Results

### Cohort characteristics

The study included 124 patients from 205 initially assessed (inclusion rate: 60.5%). Among these, 99 patients (79.8%) survived and 25 (20.2%) died.

#### Demographics and baseline characteristics.

The population was predominantly male (59.7%, male-to-female ratio 1.48) with median age 58.6 years [IQR: 46.3–67.2]. Age distribution showed predominance of the 60–69 years group (30.6%) and 50–59 years group (24.2%). Comorbidities were present in 65.3% of patients, with arterial hypertension being most frequent (50%), followed by diabetes mellitus (29.8%) and obesity (21%).

#### Clinical presentation.

At admission, initial symptomatology showed clear predominance of respiratory signs: cough (73.4%), dyspnea (70.2%), and fever (68.5%) constituted the main symptomatic triad. Other frequent signs included fatigue (58.1%), arthralgias or myalgias (56.5%), and headaches (41.1%). Vital signs revealed significant abnormalities: median SpO_2_ was 88% [IQR: 82–91], indicating substantial hypoxemia in the majority of patients. Median respiratory rate was 26 bpm [IQR: 23–30], heart rate 92 bpm [IQR: 85–104], systolic blood pressure 125 mmHg [IQR: 110–140], and temperature 37.1°C [IQR: 36.6–38.2]. Clinical examination revealed pneumonia in 61.3%, respiratory distress in 18.5%, cardiac decompensation in 13.7%, and altered consciousness in 8.1%. Severity assessment using qSOFA showed 68.5% of patients with score ≥ 2, including 10.5% with maximum score of 3, indicating high baseline severity in this hospitalized cohort.

#### Laboratory and radiological findings.

Laboratory data showed median (all values are medians with IQR): CRP 48 mg/L [IQR: 12–114], D-dimers 782 ng/mL [IQR: 441–1784], lymphocytes 1.3 G/L [IQR: 0.8–2.0], neutrophils 7.4 G/L [IQR: 4.9–12.0], WBC 9.85 G/L [IQR: 7.0–15.4], glucose 7.95 mmol/L [IQR: 6.1–13.5], creatinine 98 μmol/L [IQR: 78–121], and urea 6.9 mmol/L [IQR: 5.0–10.0]. Chest CT scans were performed in 62.3% of patients (n=77). Among these, 53.2% showed bilateral involvement, with lesions predominantly peripheral (34.2%), subpleural (28.6%), and basal (18.2%). Severe lung involvement (affecting 50–75% of parenchyma) was observed in 41.6% of cases. Ground-glass opacities (59.7%) were the most frequent lesion type, followed by reticular opacities (25.8%) and consolidations (24.2%).

#### Clinical outcomes.

During hospitalization, 80.6% of patients developed at least one complication. The most frequent were respiratory deterioration (32.3%), occurring at median 3.5 days [IQR: 3–6], and diabetic decompensation (29.0%), occurring at median 3 days [IQR: 2–7]. The overall in-hospital mortality was 20.2%, with median time to death of 8 days [IQR: 4–14] from admission. Median hospitalization duration was 12 days [IQR: 0–84] for the entire cohort, 11 days [IQR: 6–18] for survivors, and 9 days [IQR: 4–15] for non-survivors.

### Statistical implicative analysis

Statistical implicative analysis identified 156 significant implication rules (q≥0.95) linking clinical, biological, and radiological variables to target outcomes. We present the most clinically relevant rules here; The main results are provided in Supplementary S1 Files.

#### Strongest predictors of severity.

Table 2 summarizes the implication rules with highest statistical significance and clinical relevance for predicting severe outcomes.

**Table 2:** Top 10 implication rules for predicting severe outcomes.

Predictor	Outcome	q Index	Support	Confidence	Category
SpO_2_ < 90%	SEVERITY	0.989	0.581	0.847	Vital Sign
RESP DISTRESS	SEVERITY	0.985	0.177	0.909	Clinical
qSOFA = 3	DEATH	0.984	0.105	0.923	Clinical Score
BILATERAL LESIONS	SEVERITY	0.981	0.532	0.829	Radiological
CRP HIGH	SEVERITY	0.981	0.452	0.804	Biological
DIAB DECOMP	SEVERITY	0.981	0.290	0.833	Complication
HIGH RR	SEVERITY	0.978	0.597	0.824	Vital Sign
HIGH D-DIMERS	SEVERITY	0.978	0.710	0.784	Biological
LESIONS >50%	SEVERITY	0.978	0.650	0.816	Radiological
qSOFA ≥ 2	SEVERITY	0.976	0.685	0.847	Clinical Score

These results highlight the dominant role of hypoxemia (SpO_2_ < 90%), respiratory distress, and elevated qSOFA score as predictors of severe outcomes. The high confidence values (>80%) indicate that these implications are not only statistically significant but also practically reliable.

#### Critical pathways to mortality.

Analysis of implication chains revealed several critical pathways leading to death (Fig 1):

##### Main pathway:


$$\text{COMORBIDITIES}\overset{0.973\;}{\to }{\text{SpO}}_{2}<90\%\overset{0.984\;}{\to }\text{qSOFA}\geq 2\overset{0.978\;}{\to }\text{RESP DETERIORATION }$$

$$\overset{0.981\;}{\to }\text{SHOCK}\overset{0.987\;}{\to }\text{DEATH }$$

This pathway demonstrates a cascade where pre-existing comorbidities lead to hypoxemia, which triggers organ dysfunction (reflected in elevated qSOFA), progressing to respiratory failure, shock, and ultimately death. The consistently high implication indices (q>0.97) throughout this chain indicate a robust causal sequence.

##### Secondary pathways:

*Inflammatory pathway:*


$$\text{ADVANCED AGE}\overset{0.976\;}{\to }\text{LYMPHOPENIA}\overset{0.975\;}{\to }\text{CRP HIGH}\overset{0.981\;}{\to }\text{SEVERITY }$$

*Metabolic pathway:*


$$\text{DIABETES}\overset{0.978\;}{\to }\text{HYPERGLYCEMIA}\overset{0.974\;}{\to }\text{ACIDOCETOSIS}\overset{0.976\;}{\to }\text{DEATH }$$

*Thrombotic pathway:*


$$\text{BILATERAL LESIONS}\overset{0.984\;}{\to }\text{HIGH D-DIMERS}\overset{0.978\;}{\to }\text{PULM EMBOLISM}\overset{0.973\;}{\to }\text{DEATH }$$

These pathways represent distinct but interconnected mechanisms of disease progression, highlighting the multifactorial nature of COVID-19 severity.

### Critical transition variables

Table 3 identifies variables that serve as critical transition points in the implicative graph, having high centrality and out-degree, indicating their role as mediators in multiple causal pathways.

**Table 3:** Critical transition variables in the implicative network.

Variable	Graph Centrality	Out-degree	Impact Level
qSOFA ≥ 2	0.847	12	Very High
SpO_2_ < 90%	0.823	8	Very High
RESP DETERIORATION	0.796	6	High
CRP HIGH	0.781	5	High
LYMPHOPENIA	0.754	4	Moderate
HIGH D-DIMERS	0.742	4	Moderate

These variables represent key intervention targets: improving oxygenation (SpO_2_), preventing respiratory deterioration, and managing inflammatory responses could potentially interrupt multiple pathways to adverse outcomes.

### Bayesian network model

#### Network structure and dependencies.

The constructed Bayesian network (Fig 2) integrates the robust implicative relationships into a coherent probabilistic framework. The network models the following key conditional dependencies:


P(Severity|Parents)=P(Severity|qSOFA, RespDistress, Inflammation) (9)

P(Complications|Parents)=P(Complications|qSOFA, Dysfunction) (10)

P(Progression|Parents)=P(Progression|Severity, Complications) (11)

P(Death|Parents)=P(Death|Severity, Progression) (12)

The hierarchical structure reflects clinical understanding of disease progression: input variables (observable measurements) influence intermediate pathophysiological states, which in turn determine clinical outcomes. This structure facilitates interpretability by making explicit the reasoning chain from observations to predictions.

### Model performance

#### Cross-validation results.

Table 4 presents the stratified 10-fold cross-validation results for all target outcomes.

**Table 4:** Stratified 10-Fold Cross-Validation Performance.

Target Variable	Precision	Recall	F1-Score	AUC (95% CI)
Mild Severity	0.87	0.89	0.88	0.92 (0.88–0.96)
Moderate Severity	0.78	0.75	0.76	0.89 (0.84–0.94)
Severe Severity	0.82	0.80	0.81	0.94 (0.90–0.98)
Complications	0.83	0.81	0.82	0.90 (0.85–0.95)
Unfavorable Progression	0.85	0.87	0.86	0.93 (0.89–0.97)
Death	0.88	0.85	0.86	0.95 (0.91–0.99)
**Mean ± SD**	**0.84 ± 0.04**	**0.83 ± 0.05**	**0.83 ± 0.04**	**0.92 ± 0.02**

The model demonstrated excellent and consistent performance across all outcomes, with AUC values exceeding 0.89 for all targets. The narrow confidence intervals and low standard deviations indicate stable performance across different data subsets. For death prediction specifically, using an optimal probability threshold of 0.4 (determined by Youden’s index), we achieved:

- Sensitivity: 76%
- Specificity: 97%
- Positive Predictive Value: 86%
- Negative Predictive Value: 94%

The high specificity and NPV indicate the model is particularly effective at ruling out mortality risk, while the good sensitivity ensures most high-risk patients are identified.

#### Comparison with alternative approaches.

Table 5 compares our Bayesian network with three commonly used machine learning approaches on the same dataset and evaluation protocol.

**Table 5:** Performance comparison with alternative machine learning approaches.

Method	Death	Severe	Prog.	Uncertain.	Interpret.
	AUC	AUC	AUC	Handling	Level
Bayesian Network	0.95	0.94	0.93	Yes	High
Logistic Regression	0.89	0.87	0.88	Limited	High
Random Forest	0.91	0.92	0.90	No	Low
SVM (RBF)	0.87	0.85	0.86	No	Very Low

Our Bayesian network achieved superior or comparable AUC to all alternative methods while providing explicit uncertainty quantification and high interpretability through its graphical structure. Random Forest achieved competitive performance (AUC 0.91–0.92) but operates as a black box, making it difficult to understand prediction rationale or assess confidence in individual predictions. This comparison demonstrates that interpretability need not come at a substantial cost to predictive performance. For clinical applications where understanding and trust are paramount, the Bayesian network offers an attractive balance of accuracy and transparency.

#### Contextualization with published COVID-19 models.

Table 6 positions our model within the broader landscape of COVID-19 prediction models.

**Table 6:** Comparison with published COVID-19 mortality prediction models.

Study	N	Method	AUC	Features
Yan et al. [4]	485	Decision Tree	0.92	3
Banoei et al. [5]	250	ML Ensemble	0.95 (train)	21
			0.91 (valid.)	
Knight et al. [27]	35,463	4C Score	0.77	8
SOFA [28]	675	Clinical Score	0.59	6
**Current Study**	**124**	**Bayesian Net**	**0.95**	**16**

Our model’s AUC (0.95) is competitive with the best-performing models in the literature. Importantly, we achieved this performance using routinely available variables suitable for resource-limited settings. While our sample size is modest, the rigorous cross-validation and robustness testing provide confidence in the results.

The key distinction of our approach is the combination of competitive performance with explicit interpretability. Models like Yan et al.’s achieve interpretability through extreme simplicity (3 features) but may sacrifice some predictive information. Our approach maintains interpretability through explicit causal structure while leveraging more comprehensive clinical information.

#### Variable importance analysis.

Table 7 presents the ranking. The ranking aligns well with clinical understanding and the implicative analysis results. The qSOFA score, which integrates multiple physiological derangements, emerges as the most informative single variable. However, the substantial mutual information of SpO_2_ and respiratory distress independently of qSOFA suggests these variables provide additional predictive value beyond the clinical score.

**Table 7:** Variable importance for death prediction (mutual information).

Variable	Mutual Information	Rank
qSOFA	0.342	1
SpO_2_	0.298	2
Respiratory Distress	0.276	3
Systemic Inflammation	0.234	4
Age	0.187	5
CRP	0.156	6
Lymphocytes	0.143	7
Comorbidities	0.128	8
D-dimers	0.115	9
Bilateral CT Involvement	0.098	10

### Robustness analysis

#### Parameter perturbation.

Table 8 summarizes model stability under various perturbation conditions.

**Table 8:** Model robustness under parameter perturbation and input noise.

Perturbation Type	Mean Variation	Std. Dev.	Robustness Score
5% CPT perturbation	0.023	0.012	0.95
10% CPT perturbation	0.048	0.025	0.90
15% CPT perturbation	0.074	0.038	0.85
5% input noise	0.019	0.010	0.96
10% input noise	0.041	0.021	0.91
15% input noise	0.067	0.034	0.87
10% missing data	0.052	0.028	0.89
20% missing data	0.089	0.045	0.81
30% missing data	0.143	0.072	0.69

The model demonstrated satisfactory robustness to moderate perturbations. Even with 15% parameter perturbation—substantially higher than expected parameter estimation error—prediction variation remained below 8%, and the robustness score stayed above 0.85. This suggests the model would perform reliably even in settings with moderate measurement uncertainty or slight miscalibration. The model showed greater sensitivity to missing data (as expected), with robustness degrading more substantially when 30% of input variables were unavailable. This highlights the importance of complete data collection when feasible, though performance remained acceptable with up to 20% missing inputs.

#### Bootstrap validation.

Bootstrap analysis with 1,000 resamples confirmed stability of performance estimates. The 95% bootstrap confidence intervals for death prediction AUC were [0.91, 0.98], closely matching the DeLong confidence intervals, with no evidence of optimistic bias. Parameter estimates (CPT values) showed low variance across bootstrap samples (mean coefficient of variation: 12%), indicating stable parameter learning despite modest sample size.

## Discussion

### Principal findings

This study developed and validated an interpretable Bayesian network model for predicting clinical outcomes in hospitalized COVID-19 patients in a resource-limited setting. The model achieved excellent predictive performance (AUC 0.95 for mortality) while maintaining transparency through explicit representation of causal relationships. Importantly, performance was competitive with or superior to alternative machine learning approaches applied to the same dataset, demonstrating that interpretability need not compromise accuracy.

### Methodological contributions

#### Integration of statistical implicative analysis.

Our use of statistical implicative analysis to guide Bayesian network structure learning represents a novel methodological contribution. Traditional structure learning algorithms rely primarily on conditional independence tests or score-based optimization, which may identify spurious relationships or miss clinically important connections in limited datasets. By first identifying robust directional implications through SIA, we constrained the structure learning process to focus on relationships with strong statistical support and clinical plausibility.

This hybrid approach offers several advantages:

1. **Reduced search space:** SIA identifies a subset of promising edges, reducing the exponentially large space of possible network structures
2. **Incorporation of asymmetry:** Implication relationships are inherently directional, providing information about causal direction that symmetrical correlation measures cannot capture
3. **Robustness to sample size:** SIA’s focus on quasi-deterministic relationships helps identify stable patterns even in modest samples
4. **Clinical interpretability:** Implication rules have intuitive interpretation as “if-then” relationships familiar to clinicians

#### Comparison with existing literature.

Our model’s performance (AUC 0.95) compares favorably with published COVID-19 prediction models. Wynants et al. [3] reviewed 232 COVID-19 prediction models, finding that most reported C-indices ranging from 0.65 to > 0.99, but the majority suffered from high risk of bias, poor reporting, and lack of external validation. Our rigorous cross-validation and explicit treatment of uncertainty address many of these concerns.

Compared to Yan et al.’s interpretable decision tree [4] (AUC 0.92 with 3 biomarkers), our model achieves higher discrimination while using routinely available clinical variables rather than specialized biomarkers. This is advantageous for LMICs where access to advanced laboratory testing may be limited. The decision tree’s simplicity (3 features) maximizes interpretability but may miss important interactions captured by our network’s richer structure.

Banoei et al. [5] achieved training AUC 0.95 and validation AUC 0.91 using machine learning ensembles across multiple centers. While their external validation is a strength, their model operates as a black box. Our Bayesian network provides comparable performance with superior interpretability, potentially facilitating clinical adoption and trust.

Importantly, our model substantially outperforms traditional clinical scores. Raschke et al. [28] reported SOFA achieving AUC 0.59 for COVID-19 mortality in a large cohort, far below our performance. This suggests that probabilistic integration of multiple variables can significantly improve upon simple scoring systems while remaining interpretable.

### Clinical interpretability and actionable insights

#### Critical pathways and intervention targets.

The identified critical pathways provide actionable clinical insights. The main pathway (comorbidities → hypoxemia → organ dysfunction → respiratory failure → shock → death) suggests specific intervention points:

- **Early oxygen therapy:** The central role of hypoxemia (SpO_2_ < 90%) suggests aggressive oxygenation support could interrupt progression
- **Close monitoring of high-risk patients:** Patients with comorbidities and early hypoxemia require intensive monitoring
- **Preventing respiratory deterioration:** Early identification of respiratory distress and prompt escalation of respiratory support may prevent progression to shock
- **Managing inflammation:** The inflammatory pathway highlighting CRP and lymphopenia suggests immunomodulation may benefit selected patients

#### Variable importance and clinical decision-making.

The variable importance analysis (Table 7) highlights that qSOFA score, SpO_2_, and respiratory distress together capture the majority of prognostic information. This suggests that even when complete data are unavailable, focusing on these key variables can support reasonably accurate risk stratification.

In resource-limited settings where laboratory testing or imaging may be unavailable or delayed, this finding is practically valuable. A simplified prediction pathway using primarily clinical examination (qSOFA, respiratory distress) and pulse oximetry (SpO_2_)—all readily available even in basic healthcare facilities—retains substantial predictive value.

### Applicability in resource-limited settings

#### Practical implementation considerations.

Our model’s design specifically addresses challenges in LMICs:

- **Routine variables:** All input variables are obtainable through standard clinical examination, basic laboratory testing, and where available, chest imaging. No specialized biomarkers are required.
- **Interpretability:** The graphical structure and probabilistic reasoning are accessible to clinicians without advanced statistical training. This transparency facilitates trust and adoption.
- **Uncertainty quantification:** The model provides confidence intervals for predictions, helping clinicians understand reliability and make appropriately cautious decisions.
- **Computational efficiency:** Inference is fast (milliseconds per prediction) and feasible on standard computers or even tablets, requiring no specialized hardware.
- **Robustness:** The model tolerates moderate measurement error and missing data, reflecting real-world conditions in resource-limited settings.

#### Potential impact on clinical practice.

In settings like Madagascar where specialist availability is limited, interpretable prediction tools can augment clinical decision-making by:

- Helping general practitioners identify high-risk patients requiring specialist consultation or transfer
- Supporting triage decisions when ICU beds are scarce
- Providing structured communication of patient risk to families
- Guiding empiric treatment intensification before laboratory results are available

The probabilistic framework naturally supports shared decision-making by presenting risks and uncertainties rather than deterministic classifications.

### Limitations and threats to validity

#### Sample size and generalizability.

Our relatively modest sample size (n=124) limits generalizability and statistical power for detecting weak associations. While cross-validation and bootstrap analyses suggest internal validity, external validation on independent cohorts is essential before widespread clinical deployment. We particularly emphasize the need for validation across:

- Diverse geographic populations with different baseline characteristics
- Healthcare systems with varying resource levels and practice patterns
- Different time periods with evolving treatment standards

The single-center design further limits generalizability. Hospital-level factors (admission policies, treatment protocols, resource availability) may influence the relationships between predictors and outcomes.

#### Data quality and measurement error.

Several data quality limitations merit acknowledgment:

- **Retrospective design:** Data were collected for clinical rather than research purposes, potentially introducing ascertainment bias and inconsistency in variable documentation.
- **Missing data:** While variables with >30% missingness were excluded, remaining variables still had some missing values. Multiple imputation was not employed, which could introduce bias if data were not missing completely at random.
- **Measurement variability:** Laboratory measurements may have higher variability in resource-limited settings due to equipment limitations, reagent quality, or technical expertise. Our robustness testing addresses this concern to some degree but cannot fully compensate for systematic measurement bias.
- **Inter-rater reliability:** Clinical assessments (e.g., respiratory distress) were documented by multiple physicians without formal inter-rater reliability assessment. While standardized protocols were followed, some subjective variation is inevitable.
- **Imaging availability:** Only 62.3% of patients underwent chest CT, potentially introducing selection bias if imaging was performed preferentially in sicker patients. The model’s performance when CT findings are missing requires further evaluation.

We emphasize these limitations to ensure appropriate interpretation of our results and to highlight the need for validation studies with prospectively collected, high-quality data.

### Lack of variant-stratified analysis

Unfortunately, systematic variant sequencing was not available at our institution during the study period (March 2020–March 2022). This prevents us from:

- Stratifying analyses by confirmed variant
- Testing whether implicative relationships and network structure differ across variants
- Explicitly modeling variant as a covariate in predictions

This represents a significant limitation for model interpretation and future applicability. Different SARS-CoV-2 variants exhibit substantially different clinical characteristics.

### Future directions

#### External validation and multi-center studies.

The most critical next step is external validation across diverse populations and healthcare settings. We encourage researchers to:

- Apply our published model to independent cohorts with confirmed variant information
- Test whether the model structure and parameters generalize or require local adaptation
- Assess performance across different resource levels and healthcare systems
- Evaluate prospectively in real clinical workflows

We will make our model implementation publicly available (upon acceptance) to facilitate such validation efforts.

#### Dynamic Bayesian networks for temporal modeling.

Our current model provides snapshot predictions based on current data. However, COVID-19 is inherently dynamic, with patients’ conditions evolving over hours to days. Dynamic Bayesian networks [29] could model temporal evolution, potentially enabling:

- Time-series prediction of disease trajectory
- Early warning systems detecting deterioration patterns
- Personalized monitoring strategies based on predicted risk trajectories
- Assessment of treatment effects on disease progression

Such temporal modeling could provide greater clinical value than snapshot predictions, particularly for monitoring patients on general wards where early detection of deterioration is critical.

#### Integration with electronic health records.

For practical deployment, integration with electronic health record (EHR) systems would facilitate:

- Automatic extraction of input variables
- Real-time risk calculation and alerts
- Longitudinal tracking of prediction accuracy
- Continuous model updating with new data
- Clinical decision support at the point of care

In resource-limited settings where paper records remain common, tablet-based data collection tools could provide a practical intermediate solution.

#### Extension to other infectious diseases.

The methodological framework (statistical implicative analysis + Bayesian networks) is not specific to COVID-19 and could be applied to other infectious diseases prevalent in LMICs:

- Tuberculosis severity and treatment outcome prediction [30]
- Malaria risk stratification and complication prediction
- Dengue severity prediction and triage support
- Pneumonia outcome prediction in pediatric and adult populations

The emphasis on interpretability, routine variables, and uncertainty quantification addresses common challenges across these application areas.

#### Personalization and treatment effect modeling.

Current predictions are population-level, but patient-specific characteristics may modify treatment effects. Causal inference extensions of Bayesian networks [31] could potentially model counterfactual outcomes under different treatment strategies, supporting personalized treatment decisions. However, such extensions require careful consideration of confounding and would ideally use randomized trial data or rigorous observational designs.

### Broader Implications for Global Health Informatics

This study contributes to a growing recognition that appropriate technology for global health must balance sophistication with simplicity [32]. Several lessons extend beyond COVID-19:

- **Interpretability matters:** In settings where clinical expertise is limited and specialist consultation scarce, transparent models that explain their reasoning may have greater real-world impact than marginally more accurate black boxes.
- **Uncertainty quantification is essential:** Probabilistic models that acknowledge uncertainty support more nuanced decision-making than deterministic predictions, particularly important when consequences of errors are severe.
- **Robustness to imperfect data:** Models for LMICs must tolerate missing data, measurement error, and variable data quality. Robustness testing should be a standard component of model validation.
- **Local validation is critical:** Models developed in high-resource settings may not generalize to LMICs due to different disease prevalence, patient characteristics, practice patterns, and resource availability. Local validation and adaptation are essential.
- **Implementation barriers matter:** Technical performance alone does not ensure clinical impact. Successful implementation requires attention to workflow integration, user training, institutional buy-in, and ongoing support.

The COVID-19 pandemic has accelerated innovation in predictive modeling for healthcare. As we transition from emergency response to endemic management, the focus should shift from rapid model development to rigorous validation, thoughtful implementation, and systematic evaluation of real-world impact—particularly in settings most in need of decision support tools.

## Conclusions

This study demonstrates that Bayesian networks can achieve excellent predictive performance for COVID-19 outcomes (AUC 0.95 for mortality) while maintaining the transparency and interpretability essential for clinical adoption, particularly in resource-limited settings. The integration of statistical implicative analysis with probabilistic modeling provides a methodologically rigorous approach to identifying robust causal relationships and constructing interpretable prediction models from limited data. Our model’s performance is competitive with or superior to alternative machine learning approaches and substantially exceeds traditional clinical scores, while uniquely providing explicit uncertainty quantification and transparent reasoning. The identification of critical pathways (comorbidities → hypoxemia → organ dysfunction → shock → death) provides actionable insights for clinical intervention and aligns with established COVID-19 pathophysiology. However, we emphasize important limitations: modest sample size, single-center design, temporal heterogeneity spanning multiple SARS-CoV-2 variants, and data quality constraints inherent to retrospective studies in resource-limited settings. External validation across diverse populations, variant contexts, and healthcare systems is essential before widespread clinical deployment. The broader contribution of this work is demonstrating that sophisticated probabilistic models can be both accurate and interpretable, addressing the traditional trade-off between performance and transparency. This approach holds promise not only for COVID-19 but for a range of clinical prediction tasks in global health settings where interpretability, uncertainty quantification, and robustness to imperfect data are critical requirements. As healthcare systems in LMICs continue to develop digital infrastructure, interpretable probabilistic models like Bayesian networks offer a path toward evidence-based clinical decision support that enhances rather than replaces clinical judgment—supporting the broader goal of universal health coverage through improved quality of care.

## Supporting information

S1 File Main results implication rules. The file contains the main implication rules obtained after using statistical implicative analysis. (PDF)
