# Supplementary material for: Bayesian networks for predicting clinical outcomes in COVID-19 patients: A retrospective study in a resource-limited setting
Source: PLoS One. 2026 Mar 13;21(3):e0343096. doi: 10.1371/journal.pone.0343096 (PMC12987430; doi:10.1371/journal.pone.0343096)
Supplement: S1 File — (PDF) [file pone.0343096.s001.pdf]

Supplementary Materials

Bayesian Networks for Predicting Clinical Outcomes in COVID-19 Patients:
A Retrospective Study in a Resource-Limited Setting

1 S1: Main Implication Rules Analysis

1.1 Identification of Clinical Parameters Influencing Severity

The main implication rules linking vital signs to severity are summarized in (Table 1)

Table 1: Implication rules from vital signs to severity

Implication Rule	q Index	Support	Confidence
SpO_2_LOW ⇒ SEVERITY	0.989	0.581	0.847
HIGH_RR ⇒ SEVERITY	0.978	0.597	0.824
HIGH_TEMP ⇒ SEVERITY	0.973	0.258	0.781
HIGH_HR ⇒ SEVERITY	0.965	0.387	0.742
HYPOTENSION ⇒ SEVERITY	0.961	0.177	0.818

The main implication rules linking physical signs to severity are summarized in (Table 2)

Table 2: Implication rules from physical signs to severity

Implication Rule	q Index	Support	Confidence
RESPIRATORY_DISTRESS ⇒ SEVERITY	0.985	0.177	0.909
CLINICAL_PNEUMONIA ⇒ SEVERITY	0.976	0.613	0.763
CONSCIOUSNESS_ALTERATION ⇒ SEVERITY	0.982	0.081	0.900
CARDIAC_SIGNS ⇒ SEVERITY	0.978	0.137	0.824
CARDIAC_DECOMPENSATION ⇒ SEVERITY	0.971	0.105	0.846

The main implication rules linking qSOFA score are summarized in (Table 3)

Table 3: Implication rules of the qSOFA score

Implication Rule	q Index	Support	Confidence
qSOFA_3 ⇒ DEATH	0.984	0.105	0.923
qSOFA_2 ⇒ SEVERITY	0.976	0.685	0.847
qSOFA_GE2 ⇒ COMPLICATIONS	0.971	0.790	0.798

The main implication rules linking respiratory symptoms are summarized in (Table 4)

Table 4: Implication rules from respiratory symptoms

Implication Rule	q Index	Support	Confidence
SEVERE_DYSPNEA ⇒ SEVERITY	0.984	0.702	0.806
PERSISTENT_COUGH ⇒ SEVERITY	0.967	0.734	0.758
CHEST_PAIN ⇒ SEVERITY	0.962	0.210	0.769
EXPECTORATION ⇒ SEVERITY	0.956	0.226	0.714

The main implication rules linking systemic symptoms are summarized in (Table 5)

Table 5: Implication rules from systemic symptoms

Implication Rule	q Index	Support	Confidence
SEVERE_FATIGUE ⇒ SEVERITY	0.971	0.581	0.736
SEVERE_HEADACHE ⇒ SEVERITY	0.964	0.411	0.686
MYALGIAS_ARTHRALGIAS ⇒ SEVERITY	0.958	0.565	0.714
DIGESTIVE_DISORDERS ⇒ SEVERITY	0.952	0.161	0.650

1.2 Identification of Biological Parameters Influencing Severity

The main implication rules linking CRP are summarized in (Table 6)

Table 6: Implication rules of CRP

Implication Rule	q Index	Support	Confidence
CRP_HIGH ⇒ SEVERITY	0.981	0.452	0.804
CRP_VERY_HIGH ⇒ DEATH	0.976	0.194	0.708
CRP_RISING ⇒ DETERIORATION	0.973	0.323	0.775

The main implication rules linking hematological parameters are summarized in (Table 7)

Table 7: Implication rules of hematological parameters

Implication Rule	q Index	Support	Confidence
HIGH_WBC ⇒ SEVERITY	0.975	0.516	0.781
HIGH_NEUTROPHILS ⇒ SEVERITY	0.972	0.645	0.747
LYMPHOPENIA ⇒ SEVERITY	0.968	0.355	0.727
THROMBOCYTOPENIA ⇒ COMPLICATIONS	0.963	0.161	0.700

The main implication rules linking coagulation markers are summarized in (Table 8)

Table 8: Implication rules of coagulation markers

Implication Rule	q Index	Support	Confidence
HIGH_D_DIMERS ⇒ SEVERITY	0.978	0.710	0.784
VERY_HIGH_D_DIMERS ⇒ DEATH	0.971	0.242	0.733
LOW_PT ⇒ SEVERITY	0.965	0.323	0.750
COAGULOPATHY ⇒ COMPLICATIONS	0.961	0.387	0.708

The main implication rules linking renal function are summarized in (Table 9)

Table 9: Implication rules of renal function

Implication Rule	q Index	Support	Confidence
HIGH_CREATININE ⇒ SEVERITY	0.972	0.290	0.806
HIGH_UREA ⇒ SEVERITY	0.975	0.387	0.792
RENAL_FAILURE ⇒ DEATH	0.978	0.081	0.800

The main implication rules linking metabolic parameters are summarized in (Table 10)

Table 10: Implication rules of metabolic parameters

Implication Rule	q Index	Support	Confidence
HYPERGLYCEMIA ⇒ SEVERITY	0.974	0.677	0.762
DIABETIC_DECOMPENSATION ⇒ SEVERITY	0.981	0.290	0.833
ACIDOCETOSIS ⇒ DEATH	0.976	0.081	0.800

1.3 Identification of Paraclinical Parameters Influencing Severity

The main implication rules linking thoracic imaging are summarized in (Table 11)

Table 11: Implication rules from thoracic imaging

Implication Rule	q Index	Support	Confidence
BILATERAL_LESIONS ⇒ SEVERITY	0.981	0.532	0.829
LESIONS_GT50 ⇒ SEVERITY	0.978	0.650	0.816
LESIONS_GT75 ⇒ DEATH	0.973	0.234	0.724
PERIPHERAL_LOCATION ⇒ SEVERITY	0.967	0.342	0.762
BASAL_LOCATION ⇒ SEVERITY	0.963	0.182	0.714

The main implication rules linking lesion types are summarized in (Table 12)

Table 12: Implication rules from lesion types

Implication Rule	q Index	Support	Confidence
GROUND_GLASS ⇒ SEVERITY	0.976	0.597	0.784
CONSOLIDATIONS ⇒ SEVERITY	0.971	0.242	0.800
CRAZY_PAVING ⇒ SEVERE_SEVERITY	0.985	0.048	0.833
PLEURAL_EFFUSION ⇒ SEVERITY	0.968	0.081	0.800
PULMONARY_EMBOLISM ⇒ DEATH	0.973	0.008	1.000

The main implication rules linking lesion types are summarized in (Table 13)

Table 13: Implication rules from radiological combinations

Implication Rule	q Index	Support	Confidence
BILATERAL ∧ LESIONS_GT50 ⇒ SEVERITY	0.984	0.435	0.870
GROUND_GLASS ∧ CONSOLIDATIONS ⇒ SEVERITY	0.981	0.194	0.875
CRAZY_PAVING ∧ BILATERAL ⇒ DEATH	0.987	0.032	0.750

The main implication rules linking ECG abnormalities are summarized in (Table 14)

Table 14: Implication rules from ECG abnormalities

Implication Rule	q Index	Support	Confidence
T_WAVE_ABNORMALITIES $\Rightarrow$ SEVERITY	0.968	0.226	0.750
ECG_TACHYCARDIA $\Rightarrow$ SEVERITY	0.973	0.083	0.800
SUPRA_ARRHYTHMIA $\Rightarrow$ SEVERITY	0.971	0.058	0.714
LVH $\Rightarrow$ CARDIAC_COMPLICATIONS	0.965	0.042	0.800
ST_ABNORMALITIES $\Rightarrow$ CORONARY_SYNDROME	0.978	0.033	0.750

The main implication rules linking ECG combinations are summarized in (Table 15)

Table 15: Implication rules from ECG combinations

Implication Rule	q Index	Support	Confidence
TACHYCARDIA $\wedge$ T_WAVE_ABNORMALITIES $\Rightarrow$ SEVERITY	0.975	0.058	0.857
SUPRA_ARRHYTHMIA $\wedge$ LVH $\Rightarrow$ CARDIAC_COMPLICATIONS	0.970	0.042	0.800
